# Comparative study of systemic and local delivery of mesenchymal stromal cells for the treatment of chronic kidney disease

**DOI:** 10.3389/fcell.2024.1456416

**Published:** 2024-08-21

**Authors:** Emil Gregersen, Jean-Claude Kresse, Jasmine Cicek Leifing Atay, Anders Toftegaard Boysen, Peter Nejsum, Marco Eijken, Rikke Nørregaard

**Affiliations:** ^1^ Department of Clinical Medicine, Aarhus University, Aarhus, Denmark; ^2^ Department of Infectious Diseases, Aarhus University Hospital, Aarhus, Denmark; ^3^ Department of Renal Medicine, Aarhus University Hospital, Aarhus, Denmark

**Keywords:** chronic kidney disease, inflammation, fibrosis, mesenchymal stem cells, mesenchymal stromal cells (MSCs), delivery

## Abstract

Renal fibrosis, characterized by excessive extracellular matrix accumulation, leads to a progressive decline of renal function and is a common endpoint of chronic kidney disease (CKD). Current treatments primarily focus on managing underlying diseases, offering limited direct intervention for the fibrotic process. This study explores the anti-fibrotic potential of human adipose-derived mesenchymal stromal cells (MSCs) and their derived extracellular vesicles (EVs) in the context of CKD, emphasizing the effects of systemic versus local delivery methods. Preconditioned MSCs (Pr-MSCs) were treated with TNF-α and IFN-γ to enhance their immunomodulatory capabilities, and demonstrated significant anti-fibrotic effects *in vitro*, reducing mRNA expression of fibrosis markers in TGF-β stimulated HKC-8 cells. Our *in vivo* findings from a murine unilateral ureteral obstruction (UUO) model of CKD showed that local deliveries of Pr-MSCs reduced collagen deposition and increased expression of the anti-inflammatory cytokine IL-10. Systemic administration of Pr-MSCs did not show any significant effect on UUO-induced injury. In addition, EVs did not replicate the anti-fibrotic effects observed with their parent cells, suggesting that soluble proteins or metabolites secreted by Pr-MSCs might be the primary mediators of the anti-fibrotic and immunomodulatory effects. This study provides critical insights into the therapeutic efficacy of MSCs, highlighting the importance of delivery methods and the potential of preconditioning strategies in enhancing MSC-based therapies for renal fibrosis.

## 1 Introduction

Renal fibrosis is a hallmark of chronic kidney disease (CKD) and contributes to the advancement towards end-stage renal disease (ESRD). This condition involves complex molecular and cellular events, including immune-cell infiltration and fibrotic extracellular matrix (ECM) deposition, ultimately leading to a severe loss of renal function (Djudjaj and Boor, 2019; Ruiz-Ortega et al., 2020). Despite its prevalence, therapeutic options for renal fibrosis remain limited, primarily focusing on managing underlying conditions rather than targeting fibrosis directly.

Mesenchymal stromal cells (MSCs) have emerged as a promising treatment modality for renal fibrosis due to their unique immunomodulatory effects, their ability to suppress excessive inflammation and promote tissue repair (Wei et al., 2013). MSCs preconditioned with cytokines such as Tumor necrosis factor α (TNF-α) and Interferon γ (IFN-γ) have displayed enhanced therapeutic properties. Supplementing growth media with TNF-α and IFN-γ promotes the secretion of paracrine factors such as prostaglandin E2 (PGE2) and the tryptophan-degrading enzyme indoleamine 2,3-dioxygenase (IDO), improving the immunomodulatory and regenerative capacities of MSCs (François et al., 2012; Miceli et al., 2023). Moreover, the recent focus on MSC-derived extracellular vesicles (EVs) offers an alternative cell-free therapy, carrying therapeutic microRNAs (miRNAs) such as miR-let-7c and miR-451a capable of modulating gene expression in recipient cells and contributing to kidney repair (Wang et al., 2016; Zhong et al., 2018).

The delivery of MSCs and their derivatives presents a significant challenge, with systemic and local administration routes each offering distinct advantages and challenges. While systemic delivery often through intravenous injection, is less invasive and has the potential for widespread distribution, its effectiveness is limited by the entrapment of MSCs in the capillary beds of the lungs (Fischer et al., 2009). Here, MSCs have an effect by endocrine secretion or by promoting an anti-inflammatory change in the mononuclear phagocytic system after being phagocytosed (Cheng et al., 2013; Weiss and Dahlke, 2019). Conversely, local delivery ensures a higher concentration of therapeutic agents directly at the diseased site but at the cost of increased invasiveness and potential complications. Despite the preference for systemic administration in pre-clinical and clinical settings, the optimal route for delivering MSCs and MSC EVs to the kidney remains to be determined (Kresse et al., 2023).

This study aims to investigate the anti-fibrotic potential of preconditioned human adipose-derived MSCs and MSC-derived EVs in murine CKD models, focusing on comparing the therapeutic effects of systemic versus local delivery methods to the kidney. We hypothesize that local delivery of MSCs to the injured kidney provides superior protection against fibrosis compared to systemic administration. By addressing this gap, our study seeks to contribute valuable insights into developing more effective treatments for renal fibrosis.

## 2 Materials and methods

### 2.1 MSC isolation and characterization

Human adipose-derived MSCs were cultured in Minimum Essential Medium-α with no nucleosides (Gibco Thermo Fisher Scientific, Waltham, MA, United States) supplemented with 5% PLTGold Human Platelet Lysate (Mill Creek Life Sciences, Rochester, MN, United States), 2 mM L-Glutamine (Gibco Thermo Fisher Scientific, Waltham, MA, United States) and 50 units/mL Penicillin-Streptomycin (Gibco Thermo Fisher Scientific, Waltham, MA, United States). The purification of MSCs was carried out as previously described (Aabling et al., 2023). In short, adipose tissue from an adult, who underwent cosmetic surgery, was processed and washed with PBS, followed by enzymatic digestion using collagenase. The resultant stromal vascular fraction was cultured in T175 flasks and incubated at 37°C with 5% CO_2_. The culture medium was periodically changed, and cells were subcultured before reaching 100% confluency. For long-term preservation, MSCs were resuspended in CryoStor CS10 (Stemcell Technologies, Vancouver, British Colombia, Canada) and frozen at −80°C. For extended storage, cells were maintained at a temperature of −140°C. MSCs were used between passages 3 and 7 and all MSCs were derived from the same donor. MSCs underwent phenotypic characterization to confirm their identity by assessment for the presence of mesenchymal markers, including CD73, CD90, and CD105, and for the absence of hematopoietic markers as done previously (Dominici et al., 2006). MSCs were > 90% CD14^−^, CD19^−^, CD31^−^, CD45^−^, CD73^+^, CD90^+^ and CD105^+^. Adipose tissue was collected as waste material from adults undergoing cosmetic surgery. Material was collected as fully anonymized material and used according to the Guidelines on the use of biological material in health research projects (Version 1.0. 2017, paragraph 3.1.1) of the Danish Research Ethics Committees.

### 2.2 MSC preconditioning and collection of conditioned media

Preconditioning of MSCs and subsequent collection of conditioned media (CM) was carried out as follows: A total of 3 × 10^6^ MSCs were seeded in a T175 flask containing 20 mL of culture media, supplemented with 10 ng/mL each of TNF-α and IFN-γ. The following day, the media was aspirated, and the cells were rinsed twice with PBS. Subsequently, 20 mL of advanced MEM (Gibco Thermo Fisher Scientific-Waltham, MA, United States) without serum was added to the flask. After a 48 h of incubation, the CM was harvested and subjected to sequential centrifugation: first at 440 × g for 10 min at 20°C, followed by 2,000 × g for 10 min at 20°C. The resulting supernatant was then concentrated approximately 50-fold using Amicon^®^ Ultra-15 centrifugal filters with a 10 kDa molecular weight cut-off (Merck, Rahway, NJ, United States). When not immediately utilized in downstream applications, the concentrated CM was stored at −80°C for future use. For gene expression analysis, MSCs were harvested for RNA purification following the overnight incubation with TNF-α and INF-γ.

### 2.3 EV and soluble protein isolation

A total of 500 µL of concentrated CM was applied to qEVoriginal 70 nm columns (Izon Science Ltd., Christchurch, New Zealand), pre-equilibrated with PBS. After the sample had fully entered the column, an additional 2.5 mL of PBS was added, and the subsequent flow-through was discarded as void volume. The following 2.5 mL was collected as the EV fraction, while the next 1.5 mL was discarded. Subsequently, 7.5 mL was collected as the soluble protein (SP) fraction. Fractions were reconcentrated to their initial volume of 500 µL using Amicon^®^ Ultra-15 centrifugal filters with a 10 kDa molecular weight cut-off. The size and concentration of EVs were assessed using nanoparticle tracking analysis (NTA) on a Nanosight NS300 system (Malvern Panalytical, Malvern, Worcestershire, United Kingdom). Samples were diluted in 0.22 µM filtered PBS and introduced into the system at a flow rate of 20 μL/min, maintained at a constant temperature of 23°C. Particle detection was set at a camera level of 16 and captured over five 60-second intervals. Data analysis was performed using Nanosight 3.4 software with a detection threshold set at 5.

### 2.4 TGF-β induced fibrosis assay in HKC-8 cells

HKC-8 (Human Kidney Proximal Tubular Epithelial) cells were cultured in Dulbecco’s Modified Eagle Medium (DMEM; Gibco Thermo Fisher Scientific-Waltham, MA, United States), supplemented with 10% fetal bovine serum (Gibco Thermo Fisher Scientific-Waltham, MA, United States) and 50 units/mL Penicillin-Streptomycin (Gibco Thermo Fisher Scientific-Waltham, MA, United States). The cells were incubated at 37°C in an atmosphere containing 5% CO_2_ and were allowed to grow until they reached approximately 80% confluency. For the fibrosis assay, 30,000 HKC-8 cells were seeded into each well of a 12-well plate. After a 6–12 h period to allow for cell attachment, the media was replaced with serum-free culture media. The next day, media was further replaced with serum-free media containing 10 ng/mL TGF-β (Merck, Rahway, NJ, United States), either alone or in combination with concentrated CM, SP fraction, or EVs. The final concentrations of CM, SP fraction, and EVs were adjusted to be threefold higher than the original CM obtained from MSCs. After 48 h, HKC-8 cells were harvested for RNA purification.

### 2.5 Experimental unilateral ureteral obstruction (UUO) model

The experimental procedures conducted in this study were performed in accordance with the Danish National Guidelines for Animal Care and the published guidelines of the National Institutes of Health and approved by the Institutional Animal Care and Use Committee (IACUC) of Aarhus University, Department of Clinical Medicine according to the licenses for the use of experimental animals issued by the Danish Ministry of Justice (Approval number: 2020-15-0201-00617).

Male C57BL/6 mice (Janvier Labs), aged 9 weeks and weighing between 20 and 25 g, were obtained from Janvier. The mice were housed in a specific pathogen-free facility with a controlled temperature (22°C ± 2°C) and a 12 h light-dark cycle with a humidity of 55%. They had *ad libitum* access to standard rodent chow and water. The animals were randomly assigned to different experimental groups: SHAM (n = 5 for RNA and protein, n = 7 for immunostaining), UUO + subcapsular collagen (n = 6 for RNA and protein, n = 6 for immunostaining), UUO + subcapsular MSCs suspended in collagen (n = 6 for RNA and protein, n = 9 for immunostaining), UUO + subcapsular EVs suspended in collagen (n = 6 for RNA and protein), UUO + systemic saline (n = 6 for RNA and protein, n = 8 for immunostaining), UUO + systemic MSCs suspended in saline (n = 5 for RNA and protein, n = 7 for immunostaining), UUO + systemic EVs suspended in saline (n = 4 for RNA and protein).

Mice were subjected to 5 days of UUO to mimic renal fibrosis and kidney injury. Mice were anesthetized with sevoflurane (5% induction, 3%-4% maintenance) with oxygen supplementation. A midline abdominal incision was made, and the left ureter was exposed and dissected free from surrounding connective tissue. The ureter was then ligated using non-absorbable 6–0 silk sutures creating a UUO model. SHAM animals were treated similarly but the ligature around the ureter was omitted. The abdominal incision was sutured in two layers, muscle, and skin, and the mice were allowed to recover. Before surgery commenced, mice were given subcutaneous injections of buprenorphine (Temgesic, Indivior UK Limited, Berkshire, United Kingdom), which additionally was provided in the drinking water for 3 days post-surgery. MSCs (3 million cells) were administered either locally in a collagen hydrogel matrix or via the tail vein in saline, depending on the experimental group. For the preparation of EVs, EVs were isolated from CM from MSCs (3 million). For local administration, MSCs or EVs were suspended in 30 µL collagen I solution (Corning, Corning, NY, United States). The collagen hydrogel solution was prepared by mixing collagen 9:1 with 10xPBS and adjusting the pH to 7. The collagen solution was kept on ice until injection under the capsule during the UUO procedure. Upon injection under the capsule, the collagen hydrogel matrix transitions from a liquid to a gel as the temperature increases from approximately 4°C to body temperature (38°C) *in situ*. For systemic administration, MSCs (3 million cells) were resuspended in 100–200 µL of sterile saline and manually injected via the tail vein using a 27-gauge syringe prior to the induction of UUO. Control groups received collagen alone or saline alone using the same administration routes. The mice were monitored and weighed daily as well as checked for general health and any signs of distress. Five days after UUO induction and MSC administration, a blood sample was collected for analysis of plasma creatinine using a Creatinine Assay Kit (Merck, Rahway, NJ, United States), according to the manufacturer’s instructions. Kidneys were harvested and snap-frozen in liquid nitrogen for further analysis and mice were euthanized by cervical dislocation. For histological and immunohistochemical analysis, mice were perfused with PBS followed by 4% paraformaldehyde (PFA) through the left ventricle. Subsequently, the kidney was dissected and incubated in 4% PFA overnight.

### 2.6 RNA and DNA isolation, cDNA synthesis, and qPCR

RNA was isolated from HKC-8 cells using TRIzol Reagent (Life Technologies, Thermo Fisher Scientific, Waltham, MA, United States) and from kidney tissue samples using the NucleoSpin RNA II mini kit (Macherey Nagel, Düren, Germany), both according to manufacturers protocols. RNA concentration was quantified via spectrophotometry and stored at −80°C. cDNA synthesis was carried out using the RevertAid First Strand Synthesis Kit (Thermo Fisher Scientific, Waltham, MA, United States). DNA was isolated from kidney tissue and MSCs with the DNeasy Blood and Tissue Kit (Qiagen, Hilden, Germany) according to the manufacturer’s protocol. To provide a standard curve for detection of MSC DNA in kidney tissue, serial dilutions of DNA isolate from MSCs and sham kidney tissue was carried out and analyzed. For qPCR analysis, Brilliant SYBR Green qPCR Master Mix (Merck, Rahway, NJ, United States) was combined with gene-specific forward and reverse primers (Table 1). For both DNA and cDNA, the amplification process was executed using an Aria Mx3000P qPCR System (Agilent Technologies, Santa Clara, CA, United States). The specificity of the mouse gene-specific primers was verified using human and murine tissue (Supplementary Figure S1).

**TABLE 1:** Primer.

Gene	Forward primer 5′-3′	Reverse primer 5′-3′
Human gene primers		
αSMA	CTG​ACA​GAG​GCA​CCA​CTG​AA	TAC​ATG​GCT​GGG​ACA​TTG​AA
Col1α1	CAC​CCT​CAA​GAG​CCT​GAG​TC	ACT​CTC​CGC​TCT​TCC​AGT​CA
FN	CAG​TGG​GAG​ACC​TCG​AGA​AG	GTC​CCT​CGG​AAC​ATC​AGA​AA
IL-6	CAT​CCT​CGA​CGG​CAT​CTC​AG	TCA​CCA​GGC​AAG​TCT​CCT​CA
IL-8	AGC​TCT​GTG​TGA​AGG​TGC​AG	CCA​GTT​TTC​CTT​GGG​GTC​CA
IL-1RA	CCT​CCG​CAG​TCA​CCT​AAT​CA	TCC​CAA​GAA​CAG​AGC​ATG​AGG
VEGF	ACT​GCC​ATC​CAA​TCG​AGA​CC	TAT​GTG​CTG​GCC​TTG​GTG​AG
LIF	ATA​CGC​CAC​CCA​TGT​CAC​AA	GTC​ACG​TTG​GGG​CCA​CAT​A
Mouse gene-specific primers		
αSMA	TCC​TCC​TTT​GGC​CAA​CAT​CC	ACA​CCC​TTG​GCT​TCC​TCA​TC
Col1α1	CAA​TGG​TGA​GAC​GTG​GAA​ACC	ACA​GTC​CAG​TTC​TTC​ATT​GCA
FN	CCT​CTG​CTC​TTG​GGG​CTC​A	AGT​GGA​TGG​GAG​GAG​AGT​CG
Mouse gene primers		
IL-6	GAT​GCT​ACC​AAA​CTG​GAT​ATA​ATC	GGT​CCT​TAG​CCA​CTC​CTT​CTG​TG
TNFα	AGGCTGCCCCGACTACGT	GAC​TTT​CTC​CTG​GTA​TGA​GAT​AGC​AAA
IL-1β	CAG​GCA​GGC​AGT​ATC​ACT​CA	TGT​CCT​CAT​CCT​GGA​AGG​TC
IL-10	CCA​GTT​TTA​CCT​GGT​AGA​AGT​GAT​G	TGT​CTA​GGT​CCT​GGA​GTC​CAG​CAG​ACT​CAA
ALU assay specific primers		
ALU (human)	CAT GGT GAA ACC CCG TCT CTA	GCC TCA GCC TCC CGA GTA G
Actin (mouse)	GAT GCA CAG TAG GTC TAA GTG GAG	CAC TCA GGG CAG GTG AAA CT

### 2.7 Western blotting

For western blot analysis of EVs, both the CM and the EV fraction underwent ultracentrifugation at 140,000 × g for 2 h at 4°C. The supernatant was subsequently discarded, and the resulting EV pellet was resuspended in PBS. This resuspended pellet was then subjected to a second round of ultracentrifugation under the same conditions. Finally, the EV pellet was resuspended in RIPA buffer (50 mM Tris-HCl, 150 mM NaCl, 1 mM Na-EDTA, 1% Triton X-100, 0.5% sodium deoxycholate, pH = 7.4), supplemented with Complete™ protease inhibitors (Roche Diagnostics, Rotkreuz, Switzerland). For MSCs, these were lysed in M-PER (Thermo Fisher Scientific, Waltham, MA, United States) supplemented with phosphatase inhibitors 2 and 3 (Merck, Rahway, NJ, United States) and a protease inhibitor cocktail tablet (Roche Diagnostic, Rotkreuz, Switzerland). Next, protein samples were denatured using a loading buffer containing 2% SDS and DTT, and incubated at 65°C for 15 min. The denatured proteins were separated on a 12% Criterion TGX Stain-Free gel and transferred to a nitrocellulose membrane. The membrane was blocked with a 5% skimmed milk solution in PBS-Tween and subsequently washed. It was then incubated with specific primary antibodies, as listed in Table 2. After a second round of washing, the membrane was incubated with the corresponding secondary antibody. Protein bands were visualized using ECL-Prime detection reagent (GE-healthcare, Chicago, Il, United States) and captured with a Western Blot Imager (ChemiDoc MP, Bio-Rad, Hercules, CA, United States). Protein expression levels were normalized to total protein content.

**TABLE 2:** Antibodies.

Target	Antibody	Dilution
HNA	Abcam – ab190710	250
αSMA	Dako – M0851	1,000
CD63	Abcam – ab134045	1,000
TSG101	Abcam – ab125011	1,000
Calnexin	Abcam – 133615	1,000

### 2.8 Immunohistochemistry

After thorough rinsing with PBS, dehydration was achieved by utilizing a series of alcohol solutions and afterward, the tissue was embedded in paraffin. The resulting deparaffinized and rehydrated tissue sections (2 and 5 μm) were then subjected to a blocking step using a solution of 35% hydrogen peroxide (H_2_O_2_) in methanol for a duration of 30 min. Epitope retrieval was performed by boiling the sections in TEG buffer (1 mM Tris, 0.5 mM ETA, pH 9.0), followed by cooling and subsequent blocking with 50 mM NH4Cl in PBS. Next, the sections were incubated with HNA or FN antibody (Table 2) diluted in a solution of PBS containing 0.1% BSA and 0.3% Triton X100. This incubation process was carried out for 1 hour at room temperature within a humidity chamber, followed by overnight incubation at 4°C. After three successive washes with a PBS solution containing 0.1% BSA, 0.05% saponin, and 0.2% gelatin, the sections were incubated with a secondary antibody (P448, diluted 1:300 in washing solution) for 1 hour at room temperature. Subsequently, the sections underwent three additional washes using the washing solution, and visualization of the antibody-antigen reactions was achieved by incubating the sections with 3,3′-diaminobenzidine tetrachloride (DAB) dissolved in water containing 0.1% H_2_O_2_. Kidney tissue sections were scanned using the Olympus VS120 Virtual Slide Scanner. Additionally, collagen deposition was visualized using Picro Sirius Red. Quantification involved quantifying and measuring the fibrotic area as a percentage of the total area in a blinded manner from the whole kidney tissue sections. Here, we omitted the outermost cortex to minimize the chance of including MSCs and their ECM in the quantifications as well as omitting the inner medulla using QuPath version 0.5.0.

### 2.9 αSMA immunofluorescence staining

To perform immunofluorescence labeling, sections were initially incubated with a mouse-on-mouse blocking solution containing unconjugated AffiniPure Fab Fragment Donkey Anti-Mouse IgG (Jackson ImmunoResearch, West Grove, PA, United States) in PBS for 1 hour at room temperature. Following this step, the sections were post-fixed for 10 min in 4% PFA. Subsequently, an overnight incubation at 4°C with the primary antibody against αSMA (Table 2) diluted in PBS containing 0.1% BSA and 0.3% Triton X100 was performed. After a 30-min wash with PBS containing 0.1% BSA, 0.2% gelatin, and 0.05% saponin, the sections were incubated with an Alexa Fluor 488-conjugated secondary antibody (Life Technologies, Carlsbad, CA, United States) at room temperature for a duration of 30 min. Counterstaining was achieved using 4,6-diamindino-2-phenylindole (DAPI), followed by rinsing the sections with PBS and mounting them with SlowFade Gold Antifade Mountant (Life Technologies, Carlsbad, CA, United States). Whole kidney scan fluorescent images were obtained using an Olympus VS120 Virtual Slide Scanner. Quantification was carried out using ImageJ (Fiji) on selections of whole kidney scans, omitting outermost cortex as well as the inner medulla.

### 2.10 Cytokine measurements

Cytokine levels in plasma and kidney lysate from mice were assessed using the V-PLEX Mouse Cytokine 19-plex kit (Meso Scale Discovery, Rockville, MD, United States) according to the manufacturer’s protocol. Kidney tissue was homogenized in RIPA buffer (50 mM Tris-HCl, 150 mM NaCl, 1 mM Na2EDTA, 1% Triton X-100, 0.5% sodium deoxycholate, pH 7.4) using a TissueLyzer LT (Qiagen, Hilden, Germany). Subsequently, samples were centrifuged at 1,000 × g and the resulting supernatant was collected and the protein concentration was hereafter measured with the Pierce BCA Protein Assay kit (Thermo Fisher Scientific, Waltham, MA, United States). Measurements were performed on 50 µg of tissue lysate. Plasma samples were diluted twofold or fourfold according to protocol. The chemifluorescent signal was analyzed using the Meso QuickPlex SQ 120 platform (Meso Scale Discovery, Rockville, MD, United States). Cytokines with signals below the background were omitted from further analysis.

### 2.11 Statistical analysis

Statistical analysis was performed using GraphPad Prism software (version 10). Data were tested for normality and otherwise, data was transformed. Multiple comparisons between experimental groups were performed using a one-way ANOVA, followed by Tukey’s multiple-comparisons test. Statistical tests are specified in the figure legends. *p* < 0.05 was considered significant. Data are presented as mean ± standard error of the mean (SEM) unless otherwise stated.

## 3 Results

### 3.1 Inflammatory preconditioning potentiates the anti-fibrotic potential of MSCs

**FIGURE 1:**
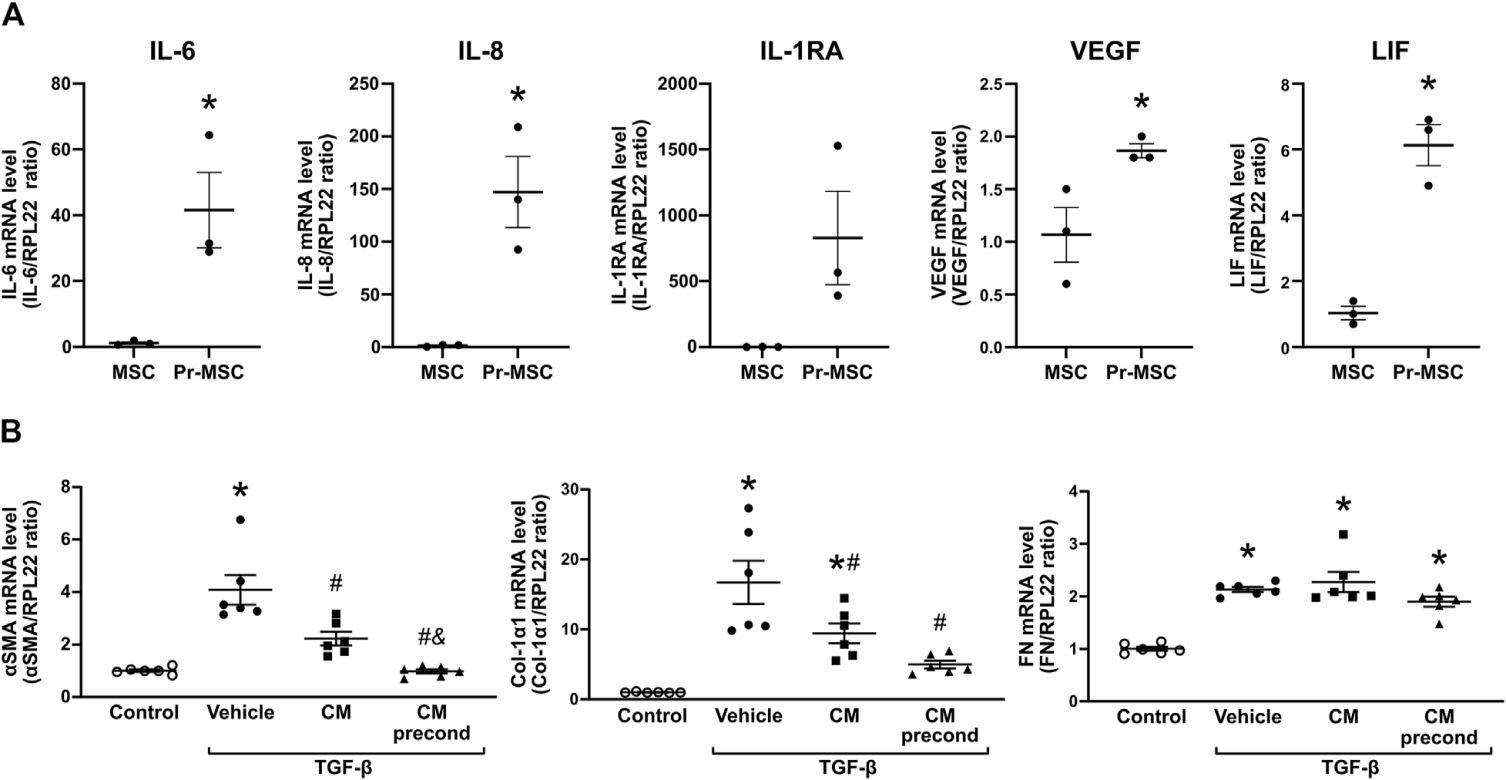
Mitigating TGF-β-induced fibrosis in HKC-8 cells by conditioned media (CM) from adipose-derived mesenchymal stromal cells (MSCs). **(A)** Gene expression analysis of MSCs, that have been preconditioned (Pr-MSC) overnight with 10 ng/mL of tumor necrosis factor-α (TNF-α) and interferon-γ (IFN-γ) or left without preconditioning. Expression levels were normalized to RPL22 and compared to the baseline expression. Statistical analysis: Unpaired t-test. *P < 0.05. **(B)** HKC-8 cells were allowed to adhere and subjected to overnight serum-free incubation. Subsequently, fibrosis was induced with transforming growth factor-β (TGF-β). Concurrently, cells were treated for 48 h with CM from MSCs without preconditioning, MSCs preconditioned with TNF-α and IFN-γ (CM precond), or a vehicle control (advanced MEM). Gene expression levels of α-smooth muscle actin (αSMA), Collagen 1a1 (Col-1a1), and Fibronectin (FN) were evaluated using qPCR and normalized to the control gene RPL22. Statistical analysis: One-way ANOVA followed by Tukey’s multiple comparison. *P < 0.05 vs. control, ^#^P < 0.05 vs. vehicle, ^&^ *p* < 0.05 vs. CM. Data are presented as mean ± SEM.

Preconditioning of MSCs, using a combination of TNF-α and IFN-γ was carried out to demonstrate the capability of this cytokine combination to increase the expression levels of several genes involved in modulating inflammatory responses and inhibiting fibrosis, including interleukin (IL)-6, IL-8, IL-1RA, vascular endothelial growth factor (VEGF), and leukemia inhibitory factor (LIF) (Figure 1A). Next, the anti-fibrotic effect of MSC-secreted factors was tested using a model of TGF-β induced fibrosis in human proximal tubule (HKC-8) cells. HKC-8 cells were incubated in the presence of CM of non-preconditioned and preconditioned MSCs (Pr-MSCs), followed by analysis of the fibrosis-related genes α-smooth muscle actin (αSMA), collagen type 1 α1 chain (Col1α1), and fibronectin (FN) (Figure 1B). CM from MSCs cultured in both conditions resulted in a decrease in αSMA and Col1α1 mRNA expression in TGF-β-treated HKC-8 cells (Figure 1B). This effect was more pronounced when CM from pr-MSCs was used (Figure 1B), suggesting that preconditioning of MSCs can enhance their anti-fibrotic potential.

### 3.2 Soluble proteins and not EVs carry the anti-fibrotic and immunomodulatory effect of MSCs

**FIGURE 2:**
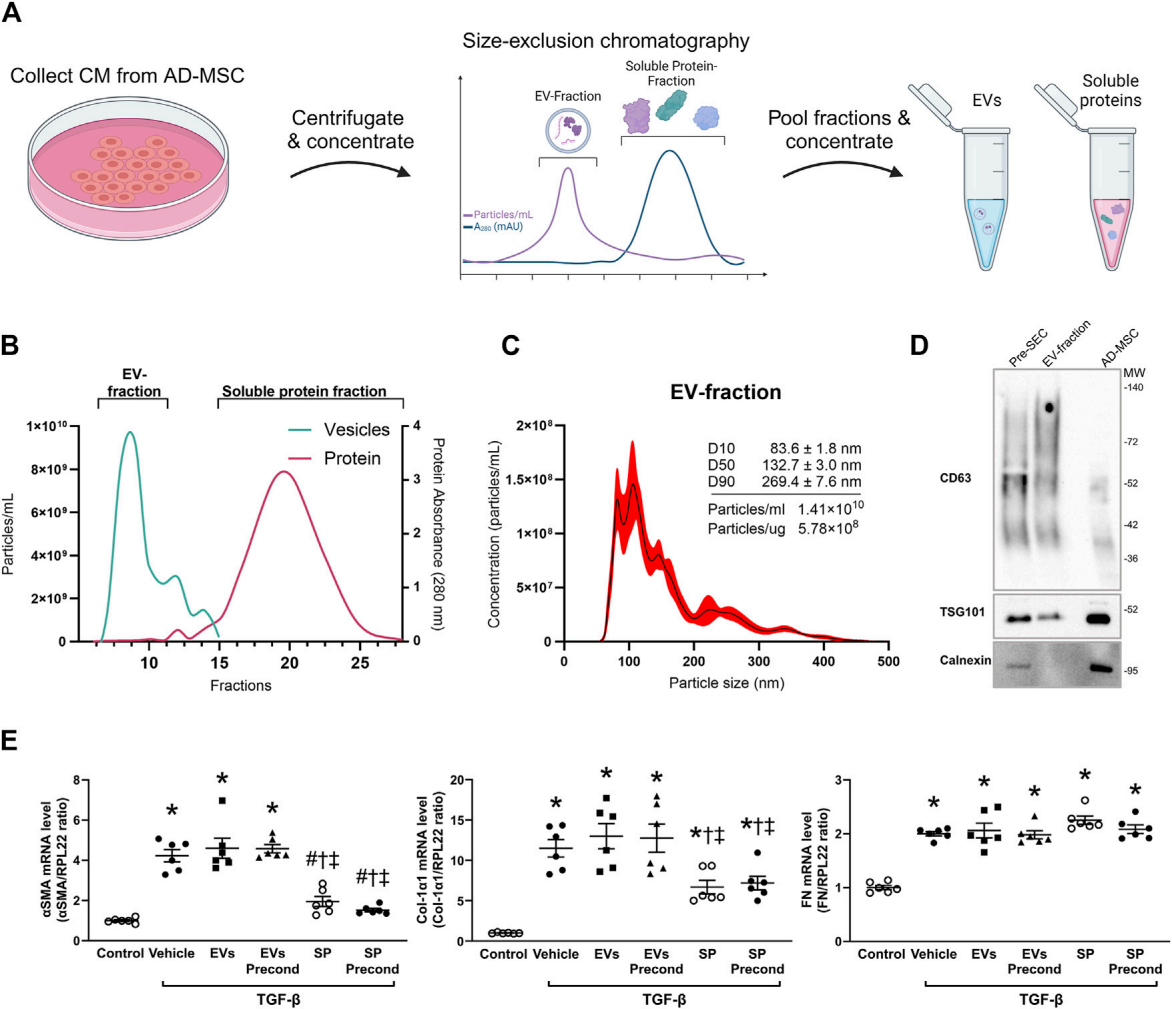
Isolation and characterization of extracellular vesicles (EVs) and soluble proteins (SPs) from adipose-derived mesenchymal stromal cells (AD-MSC). **(A)** Schematic representation depicting the isolation of EV and soluble protein isolation from conditioned media (CM) of MSCs using size-exclusion chromatography (SEC). Following SEC, both the EV and soluble protein fractions were concentrated to the same volume, resulting in the same concentration factor. **(B)** Actual elution profile of EVs and soluble proteins post-SEC, utilizing a qEVoriginal 70 nm column. Fractions of 0.5 mL were collected after the void volume (3 mL). Nanoparticle tracking analysis (NTA; depicted by the green line) was employed to estimate particle numbers, whereas protein concentrations (indicated by the red line) were quantified through absorbance measurements at 280 nm. Fractions 6–10 were combined to form the EV-enriched fraction, while fractions 14–29 were pooled as the soluble protein fraction. **(C)** Particle size and concentration of EV-fraction was analyzed using NTA. The black line represents the mean concentration, while the red line indicates the standard error of the mean. In this example, EV-fraction was concentrated around 100 times compared to CM collected from MSC. **(D)** Western blot analysis assessing the presence of CD63, TSG101, and calnexin in both EV and MSC lysates. EV lysates were acquired from EVs isolated by ultracentrifugation of CM prior to SEC, as well as from the EV-fraction obtained post-SEC. **(E)** HKC-8 cells were allowed to adhere and subjected to overnight serum-free incubation. Subsequently, fibrosis was induced with transforming growth factor-β (TGF-β). Concurrently, cells were treated for 48 h with EVs or SPs from MSCs without preconditioning, MSCs preconditioned with TNF-α and IFN-γ (precond), or PBS as vehicle control. Gene expression levels of α-smooth muscle actin (αSMA), Collagen 1α1 (Col-1α1), and Fibronectin (FN) were evaluated using qPCR and normalized to the control gene RPL22. Statistical analysis: One-way ANOVA followed by Tukey’s multiple comparison. *P < 0.05 vs. control, ^#^P < 0.05 vehicle, ^†^P < 0.05 vs. EVs, ^‡^P < 0.05 vs. precond EVs.

To identify the specific components contributing to the observed anti-fibrotic effects, we isolated EVs and soluble proteins from normal MSC and Pr-MSC CM using size-exclusion chromatography (Figure 2A). Nanoparticle tracking analysis (NTA) of concentrated EV fractions revealed a mode size of the isolated particles of 93 ± 6 nm with a concentration of 1.4 × 10^10^ particles/mL and 5 × 10^8^ particles per µg protein (Figures 2B, C). Isolated EVs were positive for the canonical EV markers CD63 and TSG101, while being negative for the apoptotic body marker Calnexin (Figure 2D).

To compare the anti-fibrotic potential of EV fraction and SP fraction, both fractions from either normal or Pr-MSCs were applied to TGF-β treated HKC-8 cells. Gene expression analysis revealed that the SP fraction of both MSC and Pr-MSC CM decreased the expression of αSMA and Col1α1 in HKC-8 cells (Figure 2E). Conversely, the EV fractions were not able to suppress αSMA and Col1α1 expression. These results suggest that the SP fraction is the primary mediator of the anti-fibrotic effects exerted by Pr-MSCs.

### 3.3 Subcapsular delivery of Pr-MSCs in a murine UUO model

**FIGURE 3:**
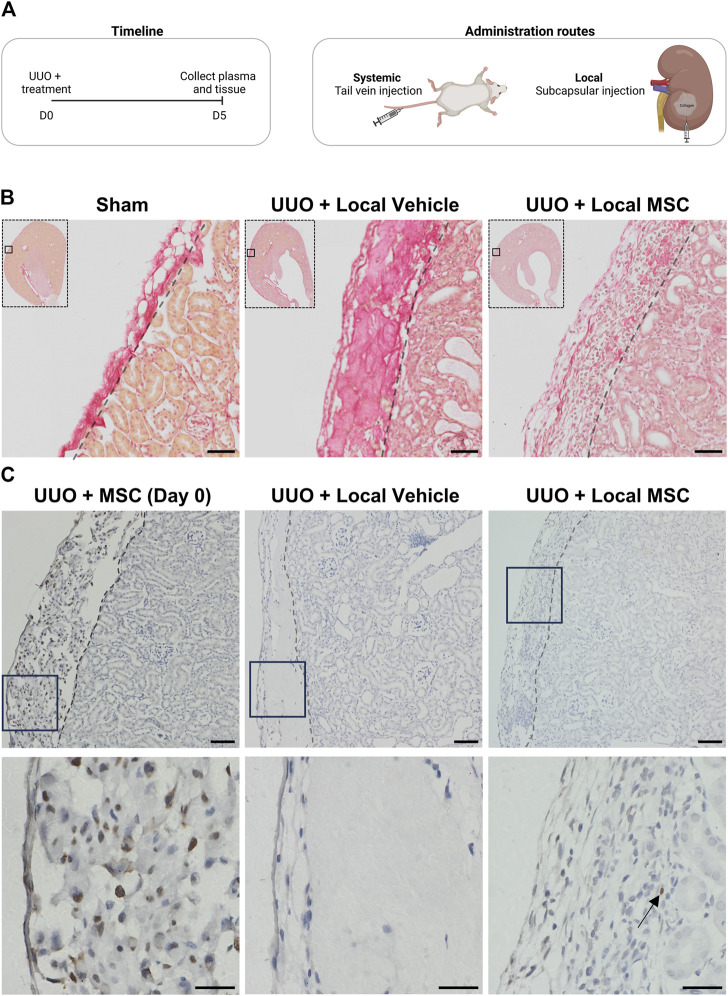
Evaluating systemic and local delivery of adipose-derived mesenchymal stromal cells (MSC) in a murine chronic kidney disease model. **(A)** Experimental timeline for murine *in vivo* study. Mice were subjected to unilateral ureteral obstruction (UUO) and received treatment on Day 0. On Day 5, the animals were euthanized, and tissue samples were collected for analysis. Two treatment routes were employed: (1) systemic administration of three million MSCs in PBS via tail vein injection, and (2) local delivery of three million MSCs embedded in a collagen hydrogel matrix through subcapsular injection as well as their vehicle alone. **(B)** Kidneys from mice subjected to UUO and treated locally with either vehicle or MSC, as well as from sham-operated mice, were assessed using Sirius red staining. Scale bar = 50 µm **(C)** HNA-stained kidney sections (x10 on top and x40 below) from UUO mice include: (1) Mice injected subcapsularly with MSCs in collagen matrix, sacrificed 3 h post-operation (Day 0), (2) UUO mice treated with subcapsular injected collagen matrix alone and harvested at day 5 (UUO + Vehicle), and (3) UUO mice injected subcapsularly with MSCs in collagen and harvested at day 5 (UUO + MSC). For x10 scale bar = 50 µm. For x40 scale bar = 20 µm. Arrows show the location of a single nucleus stained in the UUO + MSC group on day 5.

The anti-fibrotic effects of Pr-MSCs were investigated in a murine UUO model. Mice underwent 5 days of UUO and directly after induction of UUO, three million Pr-MSCs were administered either locally via renal subcapsular injection or systemically via tail vein injection (Figure 3A).

To enhance the efficacy of subcapsular local delivery, Pr-MSCs were embedded in a collagen hydrogel matrix to ensure their retention under the renal capsule. As shown in Figure 3B, Sirius red staining revealed strong staining of collagen hydrogel under the renal capsule in the vehicle-treated UUO mice. In UUO mice receiving the collagen hydrogel matrix mixed with Pr-MSC, the collagen deposition was less pronounced. Instead, an increased abundance of nucleated cells was observed, indicating the presence of cells in the collagen hydrogel after 5 days UUO. This was not observed in sham or mice subjected to systemic administration of Pr-MSCs (data not shown). Staining against the human nuclear antigen (HNA) revealed successful subcapsular delivery of human Pr-MSCs, as positive nuclei were detected in the perirenal sac between the capsule and kidney stroma 3 h post-injection, and by day five, we were still able to detect some HNA-positive MSCs (Figure 3C). In addition, we observed strong staining of macrophages under the capsule and our ALU sequence analysis did not show any engraftment of the MSCs in the renal tissue (Supplementary Figure S3).

### 3.4 Pr-MSCs ameliorate fibrotic collagen deposition in a murine UUO model

Next, we evaluated the therapeutic efficacy of local renal subcapsular versus systemic delivery of Pr-MSCs in the UUO mouse model. Six mice were lost due to surgical difficulties. To determine the effects of Pr-MSCs treatment on body weight and renal function in UUO mice, we measured body weight, kidney weight, and plasma creatinine levels (Table 3). Throughout the experiment, the body weight of the mice remained stable, and no signs of poor health were observed. As expected, there was a noticeable increase in the weight of the left obstructed kidney when compared to sham-operated mice, and the increased kidney weight was not affected after Pr-MSC treatment. In addition, plasma creatinine levels did not exhibit any significant differences among the groups (Table 3).

**TABLE 3:** – Functional data of UUO mice.

Groups	n	Bodyweight (g)	Obstructed kidney/Bodyweight (mg/g)	Plasma creatinine (µmol/L)
Sham	5	22 ± 0.6	6.35 ± 0.2	12.3 ± 0.4
Local Vehicle	6	21 ± 0.8	8.43 ± 0.4*	13.2 ± 1.2
Local MSC	6	22 ± 0.7	9.04 ± 0.4*	13.3 ± 1.6
Local EVs	6	21 ± 1.4	8.00 ± 0.2	13.2 ± 0.9
Systemic Vehicle	6	23 ± 0.7	7.99 ± 0.2	14.0 ± 1.1
Systemic MSC	5	22 ± 0.9	9.24 ± 0.4*	14.4 ± 2.9
Systemic EVs	4	22 ± 0.02	8.40 ± 0.2*	13.0 ± 0.8

Values are presented as means ± SD *= p < 0.05 compared to sham.

**FIGURE 4:**
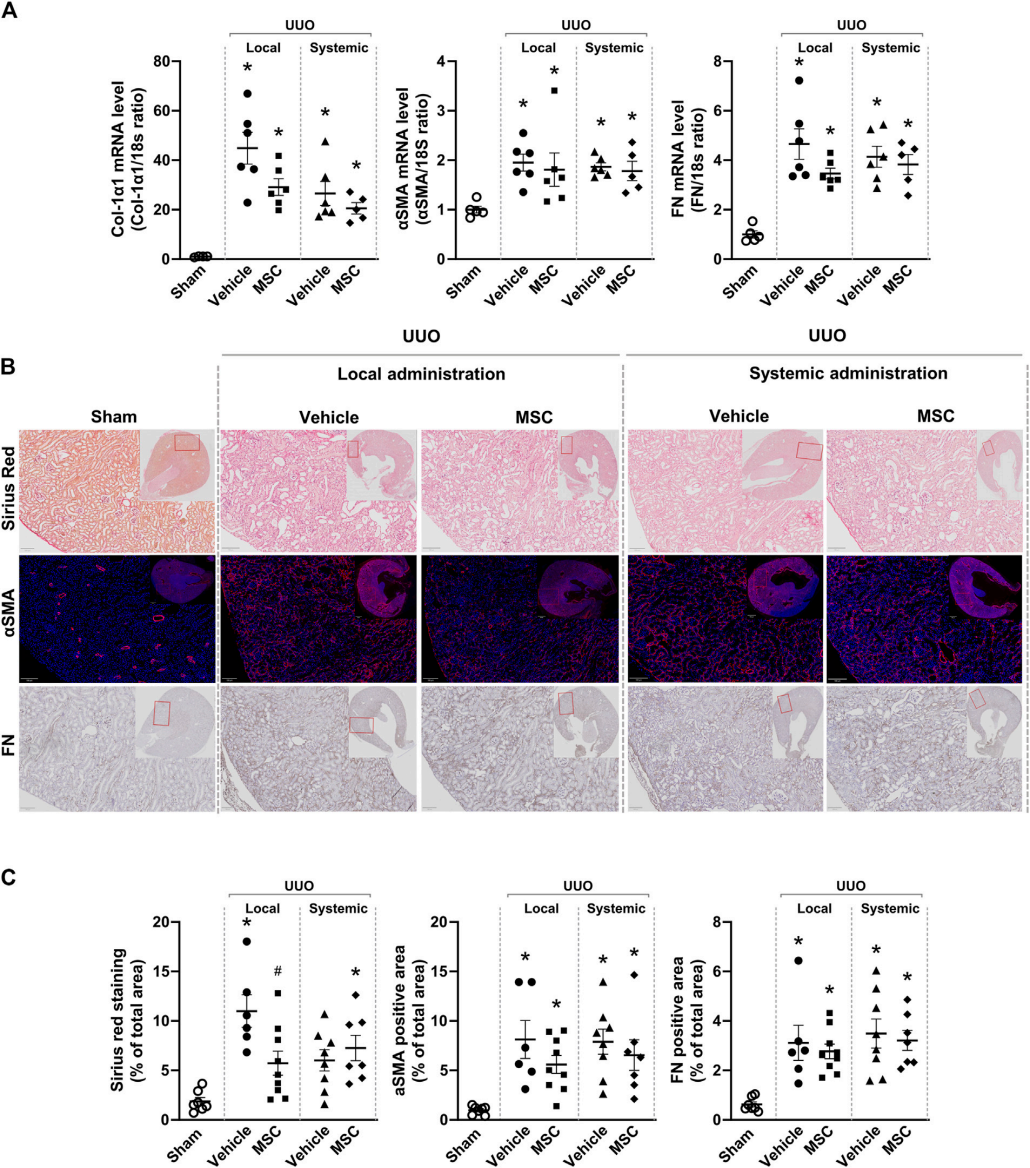
Local delivery of adipose-derived mesenchymal stromal cells (MSCs) decreases collagen deposition in mice subjected to unilateral ureteral obstruction (UUO). UUO mice received either systemic treatment via tail vein injection of MSCs or local treatment through subcapsular injection of MSCs. On Day 5 post-treatment, the animals were euthanized, and tissue samples were harvested for subsequent analysis. **(A)** Relative gene expression levels of, Collagen Type I α1 Chain (Col-1α1), αSMA, and fibronectin (FN) were measured by qPCR, with normalization to 18s expression. Statistical test: One-way ANOVA followed by Tukey’s multiple comparison. *P < 0.05 vs. sham, ^#^P < 0.05 vs. vehicle local. **(B)** Representative pictures of Sirius Red staining targeting collagen, immunofluorescent staining targeting αSMA and immunostaining of FN. Scale bar = 100 µm. **(C)** Quantification of Sirius Red stained, αSMA excluding signals originating from arterial structures and FN, on whole kidney scans excluding outermost cortex and inner medulla. Statistical analysis: One-way ANOVA followed by Tukey’s multiple comparison. *P < 0.05 vs. sham, #P < 0.05 vs. vehicle controls. Data are presented as mean ± SEM.

Using qPCR analysis, our data showed increased mRNA expression of fibrosis-related genes in mice subjected to UUO. When evaluating the effect of local versus systemic administration of Pr-MSCs in UUO mice, no significant reduction in mRNA expression of αSMA, Col1α1, or FN compared to their respective vehicle controls was observed (Figure 4A). To assess renal fibrosis in more detail on the whole kidney level, we performed Sirius Red staining as well as immunostaining of αSMA and FN (Figures 4B, C). In UUO mice, increased interstitial collagen deposition, as shown by Sirius Red staining, was significantly reduced by the local subcapsular delivery of Pr-MSCs compared to vehicle treatment. No effect was observed after systemic treatment of Pr-MSCs. In addition, immunostaining for the myofibroblast marker αSMA as well as FN was higher in UUO kidneys compared to sham. However, no significant difference in αSMA and FN staining intensity or pattern was observed after Pr-MSC treatment. Together, this indicates that compared with systemic injection, local subcapsular delivery of Pr-MSCs exhibited superior outcomes in suppressing renal collagen accumulation.

Subsequently, similar studies were performed using MSC-derived EVs. Here data showed that treatment with EVs both with systemic and subcapsular delivery did not change the expression of the fibrotic markers in UUO kidneys (Supplementary Figure S2).

### 3.5 Cytokines are regulated differently between administration routes

**FIGURE 5:**
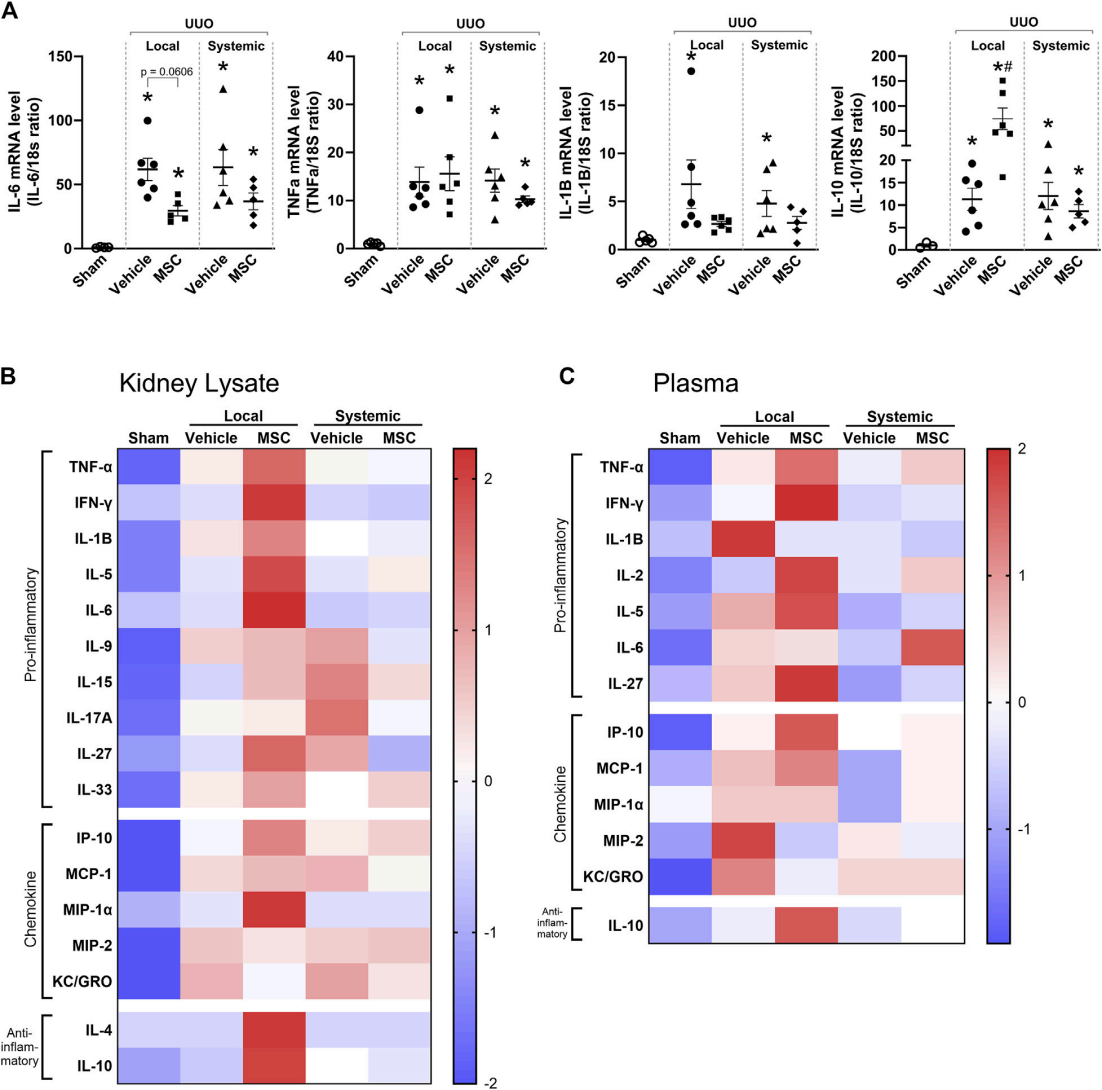
Local delivery of adipose-derived mesenchymal stromal cells (MSCs) increases pro- and anti-inflammatory factors in mice subjected to unilateral ureteral obstruction (UUO). UUO mice received either systemic treatment via tail vein injection of MSCs or local treatment through subcapsular injection of MSCs. On Day 5 post-treatment, the animals were euthanized, and tissue samples were harvested for subsequent analysis. **(A)** Relative gene expression levels of IL-6, TNF-α, IL-1β, and IL-10 were measured by qPCR, with normalization to 18s expression. Statistical test: One-way ANOVA followed by Tukey’s multiple comparison. *P < 0.05 vs. sham, ^#^P < 0.05 vs. vehicle. Data are presented as mean ± SEM. **(B, C)** Heatmap visualizing the relative protein levels of cytokines and chemokines in kidney lysate and plasma. Data is presented as z-score with the scale bar to the right indicating the range.

Cytokine expression was analyzed in Pr-MSCs treated UUO mice. UUO kidneys displayed a marked increase in IL-6, TNFα, and IL-1β mRNA levels while no difference in IL-10 was observed. Both local and systemic treatment with Pr-MSCs showed a tendency to reduce mRNA expression of IL-6 and IL-1β, but it did not reach significance. mRNA level of the anti-inflammatory cytokine IL-10 was significantly increased after local administration of Pr-MSCs (Figure 5A). A protein panel consisting of 19 cytokines and chemokines was analyzed in kidney tissue and plasma. Signals below the background were omitted from further analysis. In Figures 5B, C, the relative expression levels of all analyzed cytokines and chemokines are summarized in heatmaps. UUO mice locally injected with Pr-MSCs showed an overall pattern of increased concentrations of both pro-inflammatory, anti-inflammatory, and chemokine proteins compared to vehicle-treated UUO mice. This was seen both in kidney tissue and plasma. Effects on the cytokine profile were less pronounced after systemic administration of Pr-MSCs, indicating that the therapeutic contribution of local delivery of Pr-MSCs to renal repair may include a change in the anti-inflammatory and immunomodulating properties.

## 4 Discussion

The effect of local and systemic delivery of preconditioned human adipose-derived MSCs in a UUO animal model was investigated in this study. We find that local subcapsular delivery of Pr-MSCs in a collagen hydrogel matrix revealed superior outcomes in attenuating renal injury and collagen deposition compared to systemic administration. Our studies highlight the potential of administrating a collagen-MSC matrix in the perirenal space as an effective strategy for local delivery in kidney disease therapy.

Previous studies have shown that administering compounds in the peri space of the kidney and heart offers a viable strategy for leveraging a site-specific reservoir to achieve sustained drug release, thereby targeting organs at high local concentrations (Soranno et al., 2016; Garcia et al., 2017; Chen et al., 2021). The distinct characteristics of the perirenal sac make it an optimal platform for drug elution on the surface of the kidney, avoiding tissue damage or systemic side effects associated with intra-renal or systemic injection and ensuring uniform drug distribution throughout the kidney, even to regions distant from the injection sites. Our results reveal that the subcapsular delivery of MSCs in the collagen hydrogel matrix was well-tolerated and did not affect renal function.

Crucially, preconditioning CM derived from MSCs improved the anti-fibrotic effect of MSCs in TGF-β stimulated HKC-8 cells. Hence, we proceeded with Pr-MSCs for our *in vivo* study. We utilized human adipose-derived MSCs preconditioned with the pro-inflammatory cytokines, TNF-α and IFN-γ, and observed a significant increase in the expression of anti-inflammatory and growth factors such as IL-1β, VEGF, and LIF after preconditioning. A detrimental environment including inflammatory stimuli is known to change the secretome of MSCs (Tsuji et al., 2018) and improve their kidney protective effect (Tseng et al., 2021; Gao et al., 2022). Moreover, preconditioning primes MSCs for immediate action upon injection, bypassing the usual delay of therapeutic effect (Kanai et al., 2021).

This study used the UUO as a murine model for CKD. This model is characterized by an initial phase of inflammation followed by a phase of accumulating fibrosis which dominates the disease picture in later stages (Chevalier et al., 2009; Nørregaard et al., 2023). 5 days post surgery the kidney is in the late phase of inflammation and its early fibrotic phase. Hence, this was chosen as the optimal time point for investigating both the anti-inflammatory and anti-fibrotic effects of Pr-MSCs. Our study showed that local subcapsular delivery of Pr-MSCs reduces collagen deposition as well as increases the anti-inflammatory cytokine IL-10 in UUO-induced CKD, whereas no significant effect was observed after systemic administration. Our data is consistent with a recent study showing that subcapsular transplantation of bone marrow MSCs in a two kidneys-one clip CKD rat model exerts anti-fibrotic effects by reducing collagen deposits and increasing IL-10 expression (Almeida et al., 2022). We observed no evidence of migration of the transplanted MSCs into the kidney parenchyma, indicating that the transplanted MSCs may not differentiate within the kidney. This suggests that the therapeutic effects were due to factors secreted by the human Pr-MSCs. These results are consistent with studies on the transplantation of bone marrow MSC sheets for kidney disease, where the MSCs remained on the kidney surface and suppressed renal fibrosis through the paracrine effects of growth factors and vaso-protective cytokines (Imafuku et al., 2019).

In line with these findings, the assessment of cytokines in kidney tissue and plasma displayed a difference in the paracrine immunomodulatory effects of local and systemic delivery of Pr-MSCs. Systemic delivery induced only modest alterations in cytokine concentration compared to vehicle. In contrast, local administration of Pr-MSCs increased the concentration of multiple pro-inflammatory and anti-inflammatory factors, including IL-4 and IL-10 in kidney tissue and IL-10 in plasma. This was also reflected in increased IL-10 mRNA levels in kidney tissue. IL-10 is a key mediator of anti-inflammatory pathways which can impact renal fibrosis. For instance, knock-out of the IL-10 in a UUO mice model led to increased renal fibrosis and collagen expression (Jin et al., 2013). As MSCs can secrete IL-10 (Aggarwal and Pittenger, 2005), they could be the source of the increased IL-10 observed in the treated UUO mice. Alternatively, MSCs might affect endogenous macrophages to increase their IL-10 production (Németh et al., 2009), thereby contributing to the elevated IL-10 levels in the UUO kidneys.

The observed increase in pro-inflammatory cytokines following local Pr-MSC administration could be a response to xenotransplanted MSCs of human origin (Lin et al., 2012). These findings suggest that the paracrine mode of action of local MSC delivery plays a critical role in their therapeutic efficacy, with local application demonstrating a more pronounced impact on cytokine modulation and potential attenuation of renal fibrosis compared to systemic administration. This differential impact may be attributed to the prolonged survival of locally transplanted MSCs, which allows for a sustained release of cytokines, thereby influencing the cytokine profile observed in our study. In addition, we cannot rule out the possibility that the systemic-infused human Pr-MSCs may be entrapped in the lungs and/or cleared by the immune or complement system of the mice (Mallis et al., 2021). It has previously been demonstrated by Fischer et al. (2009), that MSCs are large cells that get rapidly entrapped in the microvasculature of the lung shortly after systemic infusion, leading to a prompt triggering of the Instant-Blood-Mediated-Inflammatory-Reaction (IBMIR). The IBMIR reaction is a cascade of innate immune reactions against the therapeutic MSC graft due to the incompatibility of the cells with human blood which may compromise cell survival and function (Caplan et al., 2019; Moll et al., 2019; Moll et al., 2022).

Several studies have demonstrated that MSC-derived EVs have reno-protective effects in preclinical AKI and CKD models (Shi et al., 2020; Tang et al., 2022). In our study, we separated EVs from MSC-derived CM by size-exclusion chromatography to identify which components of the CM mediate the anti-fibrotic effects. We noted that the anti-fibrotic properties observed in our *in vitro* model may be linked to the soluble protein fraction, rather than the EVs fraction. In addition, our *in vivo* data confirmed no impact of the MSC-derived EVs after both local and systemic administration. This supports the finding that the active components crucial for mediating the anti-fibrotic effect lie within the soluble proteins fraction of the MSC secretome. This is in line with a recent *in vitro* study that reported chromatographical separation of EVs and soluble protein, demonstrating that wound healing activity in a scratch-wound model was exclusively associated with soluble proteins and not EVs (Whittaker et al., 2020). Moreover, it has been shown that EVs do not mediate the anti-inflammatory actions of the secretome of adipose tissue-derived mouse MSCs (Carceller et al., 2021). However, the EV fraction and the soluble protein fraction can act synergistically to mediate anti-fibrotic effects, as demonstrated in a muscle injury model (Mitchell et al., 2019). The variation in outcomes observed across studies underscores the complexity of MSC-derived therapies and highlights the significant influence of MSC sources, soluble proteins, EV purification methods, and dosage on therapeutic efficacy. Future studies are needed to provide a more comprehensive understanding of the effects of soluble proteins and to explore their role *in vivo*.

Our study cannot be directly translated into a human setting. Typically, MSCs are administrated in rodents at a dose range between 50 and 250 million cells per kilogram, whereas in clinical trials the dose ranges between 1 and 10 million cells per kilogram (Hoogduijn and Lombardo, 2019; Kabat et al., 2020; Kresse et al., 2023). The dosage used in our mouse model (about 130 million cells per kilogram) was within the range used in preclinical models. The rational behind using a higher dosage in preclinical studies is to ensure a robust and observable effect with a limited experimental timeframe. Preclinical studies often use higher doses to achieve measurable outcomes and to account for differences in immune response, metabolism, and overall physiology between small animals and humans. To date, there has been no clear translation of the effective dose observed in rodents to human applications. Information on this area remains limited. However, determining the appropriate dosing regimen, including dosage and frequency, is crucial for successful translation to clinical practice (Hoogduijn and Lombardo, 2019).

Our study shows the complexity of MSC-based therapies and highlights the significant role of delivery mode on therapeutic outcomes. Local delivery of preconditioned MSCs appears to offer enhanced immunomodulatory benefits. Future research should focus on optimizing MSC delivery strategies as well as dosing and further elucidating the mechanisms underlying their therapeutic effects, to fully exploit their potential in treating chronic kidney disease.

## Data Availability

The original contributions presented in the study are included in the article/Supplementary Material, further inquiries can be directed to the corresponding author.
