# Supplementary figures and images for: Comparative study of systemic and local delivery of mesenchymal stromal cells for the treatment of chronic kidney disease
Source: Front Cell Dev Biol. 2024 Aug 21;12:1456416. doi: 10.3389/fcell.2024.1456416 (PMC11373351; doi:10.3389/fcell.2024.1456416)

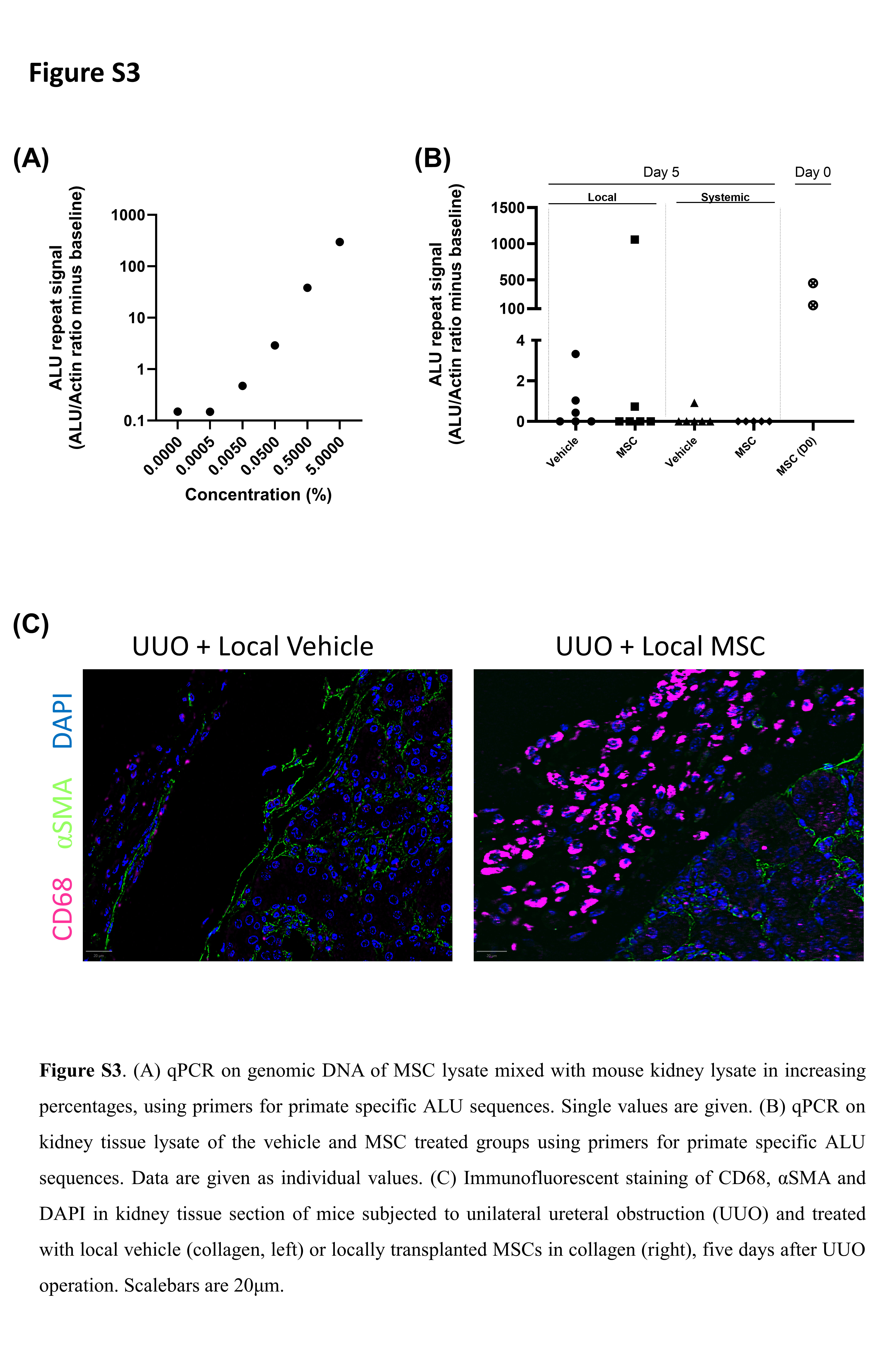

Supplement: Supplementary file 1 [file Image3.TIF]

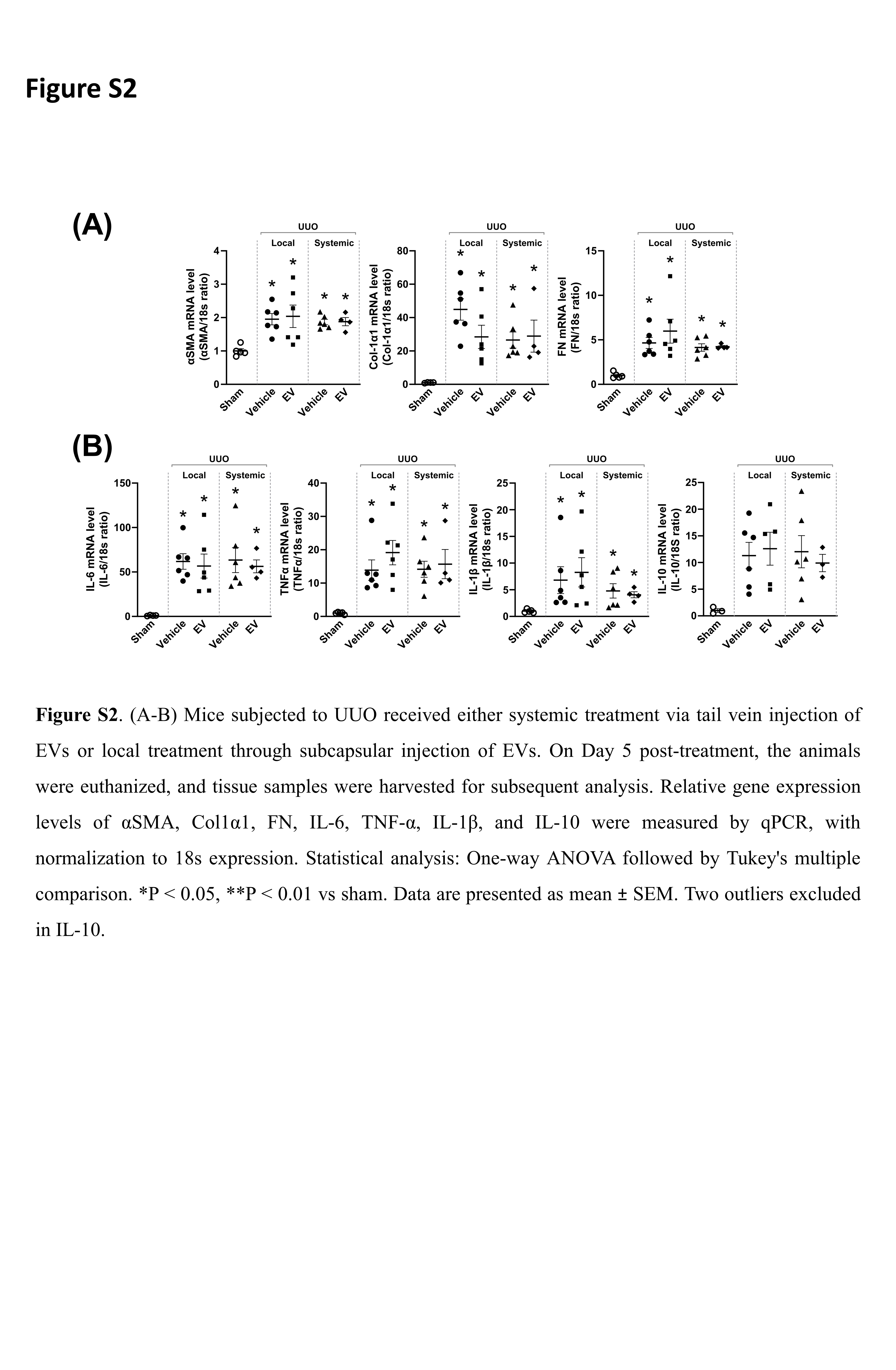

Supplement: Supplementary file 2 [file Image2.TIF]

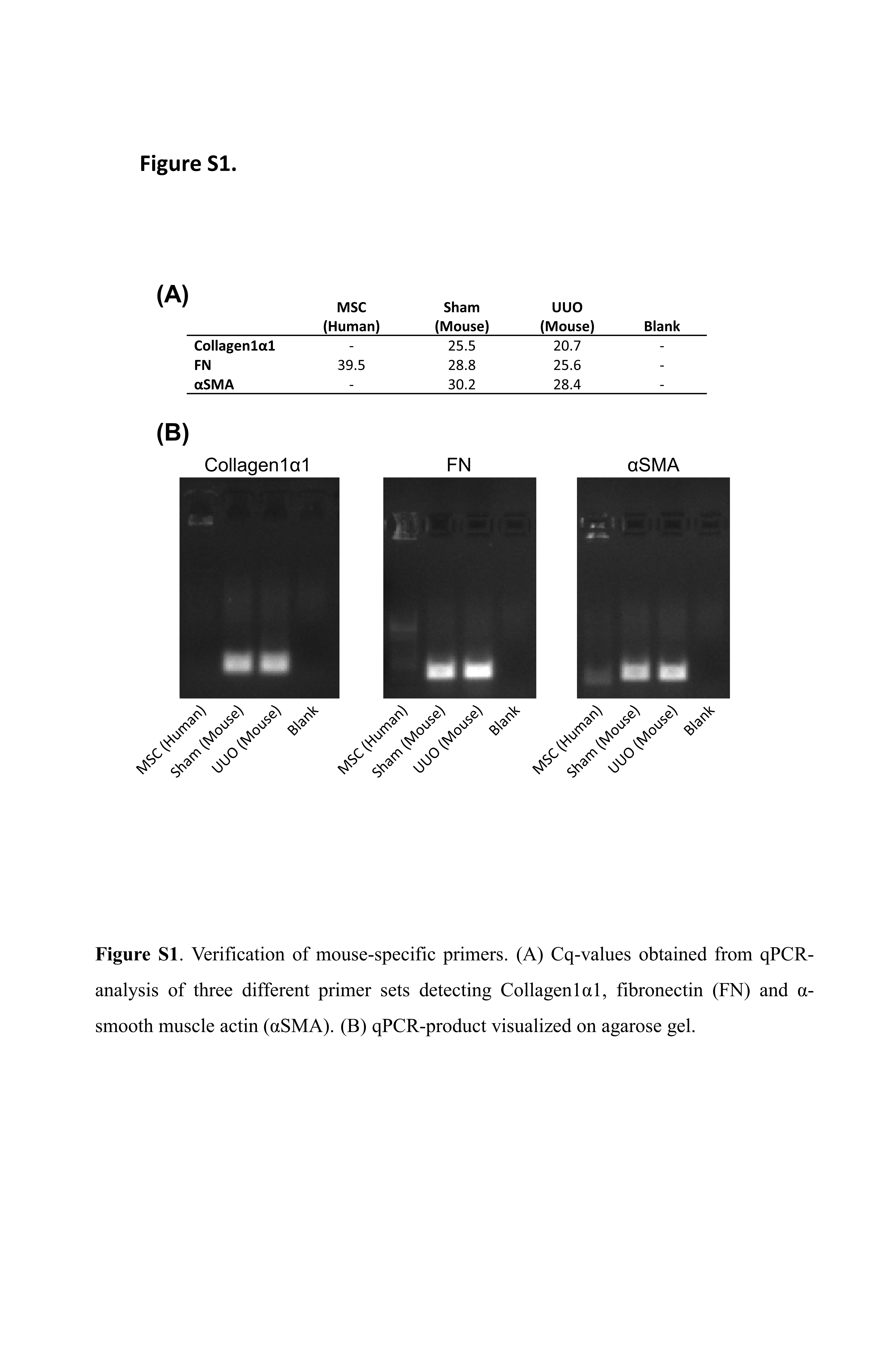

Supplement: Supplementary file 3 [file Image1.TIF]
